# Validation of an automated seizure detection algorithm for term neonates

**DOI:** 10.1016/j.clinph.2015.04.075

**Published:** 2016-01

**Authors:** Sean R. Mathieson, Nathan J. Stevenson, Evonne Low, William P. Marnane, Janet M. Rennie, Andrey Temko, Gordon Lightbody, Geraldine B. Boylan

**Affiliations:** a Neonatal Brain Research Group, Irish Centre for Fetal and Neonatal Translational Research, Department of Paediatrics and Child Health, University College Cork, Cork, Ireland; b Academic Research Department of Neonatology, Institute for Women’s Health, University College London, London, United Kingdom

**Keywords:** Neonatal seizures, Automated seizure detection, Neonatal EEG, Hypoxic-ischaemic encephalopathy, Neonatal neurology

## Abstract

**Objective:**

The objective of this study was to validate the performance of a seizure detection algorithm (SDA) developed by our group, on previously unseen, prolonged, unedited EEG recordings from 70 babies from 2 centres.

**Methods:**

EEGs of 70 babies (35 seizure, 35 non-seizure) were annotated for seizures by experts as the gold standard. The SDA was tested on the EEGs at a range of sensitivity settings. Annotations from the expert and SDA were compared using event and epoch based metrics. The effect of seizure duration on SDA performance was also analysed.

**Results:**

Between sensitivity settings of 0.5 and 0.3, the algorithm achieved seizure detection rates of 52.6–75.0%, with false detection (FD) rates of 0.04–0.36 FD/h for event based analysis, which was deemed to be acceptable in a clinical environment. Time based comparison of expert and SDA annotations using Cohen’s Kappa Index revealed a best performing SDA threshold of 0.4 (Kappa 0.630). The SDA showed improved detection performance with longer seizures.

**Conclusion:**

The SDA achieved promising performance and warrants further testing in a live clinical evaluation.

**Significance:**

The SDA has the potential to improve seizure detection and provide a robust tool for comparing treatment regimens.

## Highlights

• Seizure detection algorithm (SDA) validated on unseen, unedited EEG of 70 neonates.
• Results at SDA sensitivity settings of 0.5–0.3 acceptable for clinical use.
• Seizure detection rate of 52.6–75.0%, false detection rate 0.04–0.36 FD/h.

## 1 Introduction

The concept of “neuroprotective” intensive care has now reached neonatal units worldwide, in part driven by the results of randomized controlled trials showing that therapeutic hypothermia is beneficial for term babies with a recent hypoxic-ischaemic injury (Glass et al., 2011). The practice of neuroprotective care involves careful monitoring of carbon dioxide tension, blood pressure and other physiological variables and is ideally accompanied by continuous cotside EEG monitoring. Without EEG monitoring many seizures are missed. The inaccuracy of clinical recognition of seizures was demonstrated by Murray et al. (2008). In this study, comparing EEG evidence of seizures to the seizure detection acumen of NICU staff based on clinical evidence alone, of 526 EEG seizures, only 179 (34%) had any clinical accompaniment, overdiagnosis was common with only 48 of 177 (27%) clinically suspected events accompanied by EEG seizures such that only 48/526 (9%) of EEG seizures were correctly identified by clinical observation. Amplitude-integrated EEG (aEEG) is widely used in NICUs for seizure detection but has been shown to perform poorly (Rennie et al., 2004). In this study seizure detection by four non-experts using CFM traces at 3 paper speeds were compared against simultaneous EEG in 19 babies. Sensitivities of only 38% at 6 cm/h, 54% at 15 cm/h and 55% at 30 cm/h were achieved and agreement between observers was poor at all speeds (*κ* values from 0.01 to 0.39). Treating seizures to electrical quiescence has yet to be proven of any long-term benefit, but there is evidence from animal models (Wirrell et al., 2001), and clinical studies (Glass et al., 2009, Shah et al., 2014) which would support the principle that seizures do inflict further brain injury. Attempts to ameliorate such damage must be accompanied by prompt and reliable detection of seizures. In addition, good quality randomized controlled trials of new antiepileptic drugs are impossible without robust and reproducible EEG monitoring.

A significant barrier to the practice of neuroprotective critical care in the NICU is the lack of expertise in reporting neonatal EEG. Current cotside EEG monitors are sophisticated devices, offering the ability to record multiple channels of EEG continuously together with other physiological signals and video-recording of the baby’s movements. They allow the continuous display of aEEG and other quantitative trends and are easy to set up and maintain. But few clinicians have the knowledge to interpret the plethora of information which is generated by such monitoring, and without this knowledge there is a danger that this equipment will be under utilised or (worse) the output will be misinterpreted at the cotside.

Our group has considerable experience with cotside EEG monitoring and has grown to appreciate the benefits that this provides. For many years now we have been working on a seizure detection algorithm (SDA), which would analyse one or more channels of “raw” EEG, continuously and in real-time, providing a visual and audible alert to the clinical team. The engineering challenges have proven formidable because EEG is a complex signal, and neonatal seizures have variable amplitude, frequency and morphology, and are rarely sustained for more than 5 min.

Other groups have developed SDAs for neonates, and have published their detection rates, using varying definitions of success (Liu et al., 1992, Gotman et al., 1997, Smit et al., 2004, Navakatikyan et al., 2006, Deburchgraeve et al., 2008, Mitra et al., 2009). Details of the performance of these and other SDAs are outlined in Table 1 and reviewed further in the discussion. Currently only two SDAs are commercially available. These are the Gotman algorithm incorporated into the Stellate EEG system (Natus Medical Inc, USA); and the ‘Recognize’ algorithm of Navakatikyan which is incorporated into the Brainz aEEG monitor (Natus Medical Inc., USA) which has only a 2 channel EEG capability. One problem which inhibits comparison of SDAs is the lack of an agreed definition of what constitutes best performance. Many SDAs are reported to have good detection rates, with a high number of seizures accurately detected when compared to expert neurophysiology as the “gold standard”, and low numbers of missed seizures. However, the temporal aspect of seizure detection is rarely reported, for example one missed seizure of 8 min duration in an hour would be clinically important. Another important aspect of SDA performance assessment is the number of false detections. Many validation studies have used only short duration recordings, but any robust algorithm designed for current NICU use has to be able to perform reliably on very long recordings of 72 h or more. Respiration artefact is a particular problem often recorded in neonatal EEG and can mimic the stereotyped rhythmic seizure activity that is often seen in neonates.

We have previously reported the performance of our neonatal SDA on a set of 17 seizure babies recorded at Cork University Maternity Hospital (CUMH), Ireland (Temko et al., 2011a) using a ‘leave one out’ (LOO) cross validation method of analysis, whereby the data of one patient is used for testing and the others used for training the algorithm and the process is repeated for each patient and the mean result reported. A further LOO study was performed on 38 babies from CUMH (Temko et al., 2013) incorporating an adaptation to reduce the effects of prolonged artefact and showed improved performance. This study also incorporated analysis of an ‘unseen’ dataset of 51 babies from CUMH.

The aim of the present study was to validate the performance of our neonatal SDA on a larger database of unseen, unedited, continuous, multi-channel EEG data from 70 term newborns collected at 2 sites, CUMH and University College London Hospital (UCLH), and to provide comprehensive measures of SDA performance. While time based metrics assess the ability of the algorithm to detect the ‘amount’ of seizure activity (seizure burden) correctly and is, in a sense, the most precise engineering metric, event based metrics provide clinicians with valuable information as to the percentage of seizures that will be detected, with important implications for treatment and also how often the algorithm is likely to alarm falsely. We therefore report both time based and event based measures of performance.

## 2 Methods

### 2.1 Data acquisition and EEG annotation

Neonates were enrolled from the neonatal intensive care units of CUMH and UCLH from January 2009 to October 2011 as part of an on-going study of neonatal seizures. Neonates ⩾37 weeks gestation were enrolled for EEG monitoring if they fulfilled two or more of the following criteria: Apgar score less than six at five minutes; a continued need for resuscitation after birth; any clinical evidence of encephalopathy, or seizures developed within 72 h of age.

This study was conducted with approval from the Clinical Research Ethics Committees of the Cork Teaching hospitals, Ireland and the National Health Service in the UK, via the Integrated Research Application Service. Written, informed consent was obtained from at least one parent of each neonate who participated in this study.

#### 2.1.1 EEG recording

The EEG was recorded using a NicoletOne EEG monitor (Carefusion, Wisconsin, USA) and the 10:20 EEG electrode placement system adapted for neonates was used with the following electrodes F4, F3, T4, T3, C4, C3, CZ, O2 and O1. Additional electrodes were positioned at P3 and P4 when possible. Respiration and ECG was also monitored and signals were stored synchronously with the EEG. The EEG was recorded at a sampling rate of 250 Hz or 256 Hz, with a filter bandwidth of 0.5–70 Hz. The EEG was recorded from as soon as possible after birth and the recording continued for as long as clinically required.

#### 2.1.2 EEG analysis

All seizures were annotated on the original EEG file by a trained electrophysiologist, Sean Mathieson (SM) to generate seizure event text files for each recording. The seizure annotations of SM for all neonates were used for comparison with the SDA annotations. To verify the validity of the seizure annotations by SM, a random sample of 15/35 (42.85%) recordings with seizures were also annotated by Geraldine Boylan (GB) and compared for inter-rater reliability using Cohen’s Kappa index.

**Fig. 1:**
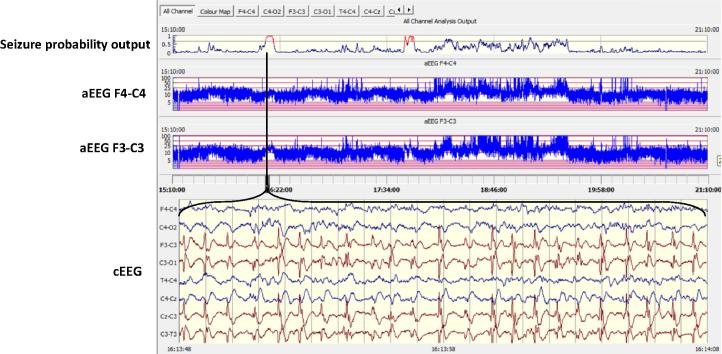
The SDA incorporated into an EEG viewer. The output of the SDA is a graph of the probability of seizure (upper panel). When a seizure is detected the trace turns red and an annotation is made. The viewer also displays the continuous EEG and aEEG.

An electrographic seizure was defined as a sudden and evolving repetitive stereotyped waveform with a definite start, middle and end, lasting for at least 10 s on at least one EEG channel (Clancy and Legido, 1987). A stand alone, offline version of the SDA was then used to process each EEG recording (see Fig. 1). Full details of the alpha version of this algorithm have been described previously (Temko et al., 2011a). The current beta version incorporates a modification to reduce false detections due to persistent artefact (Temko et al., 2013). In summary, the EEG is down-sampled to 32 Hz with an anti-aliasing filter set at 12.8 Hz and is then split into 8 s epochs with 50% overlap between epochs. Fifty-five features are then extracted for each channel from each epoch representing both time and frequency domain characteristics as well as information theory based parameters. Details of main features extracted are given in Table 2. The features extracted from each epoch are then fed into a support vector machine classifier. The output of the SDA is a graph of the probability of seizure calculated using all features in any one 8 s epoch, from zero to 1. This analysis is performed separately for each channel then results are combined for all channels into a single graph (Fig. 1, top panel). A seizure is designated when the probability graph breaches a threshold. The seizure sensitivity threshold is adjustable from 0.1 (most sensitive) to 0.9 (least sensitive). The adjustable threshold allows the algorithm to be tuned on a patient by patient basis. For example, should an EEG recording contain large amounts of artefact causing false detections, the SDA can be desensitised to reduce this number but with a concomitant decrease in the seizure detection rate, as there will always be a negative trade-off between the number of detected seizures and false detections. An SDA annotation was exported for each threshold and was used for comparison with the expert rater’s annotation. The SDA and the expert rater’s annotations were stored as text files.

### 2.2 Assessment of the SDA

**Fig. 2:**
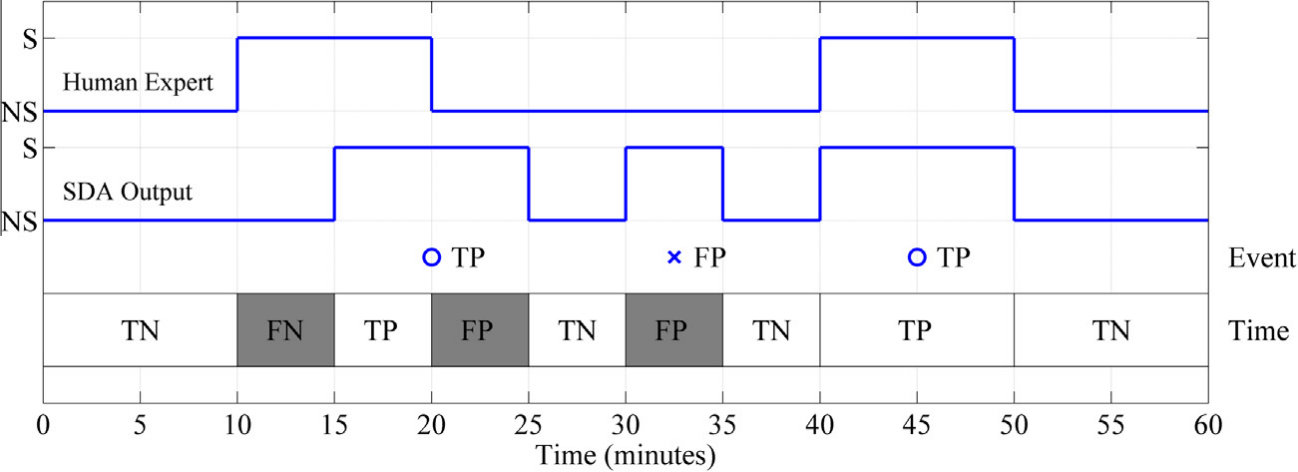
Temporal and event based assessment of agreement between the annotation of the human expert and the SDA output. S denotes seizure and NS denotes non-seizure. Light/shade in time bar denotes periods of temporal agreement/disagreement: true positives (TP), true negatives (TN), false positives (FP) and false negatives (FN). Markers denote event based agreement: TP and FP. Sensitivity for temporal assessment is 75.0% and specificity for temporal assessment is 75.0%. Sensitivity for event based assessment is 66.7% and a false alarm rate of 1/h.

Assessment of an SDA against a “gold standard” is not a trivial task (Temko et al., 2011b). There is a relative scarcity of seizures in any long duration recording, and in clinical practice recordings will be made in many babies with no seizures at all. The SDA may detect a seizure but the assessed duration might not be in agreement with the “expert” view. The possible output of a comparison is demonstrated in Fig. 2, illustrating the true positive situation (TP) when both the SDA and the expert rater agree there is seizure activity, and true negative (TN) when neither the rater or the SDA classify the EEG as showing seizure. A false positive (FP) or type 1 error occurs when the human expert did not annotate a seizure but the SDA output is seizure, and a false negative (FN) or type II error occurs when the expert annotates the recording as seizure but the SDA does not. In order to achieve a rigorous evaluation the probability output time series was set at 60 samples per minute (1 Hz). In order to measure the agreement between the annotations of seizure by the SDA and the expert rater, both records were converted into a binary time series (in this case the time series sampled at 1 Hz). The binary signal was generated by denoting the presence of a seizure at any second with ‘1’ and absence of seizure at any second with ‘0’.

#### 2.2.1 Conventional measures of agreement

Using the concept of true positive and true negative detection outlined above, conventional measures can be calculated. Sensitivity, defines agreement between the human expert and SDA for identifying the presence of seizure, TP/(TP + FN), and specificity defines agreement between the human expert and SDA for identifying the absence of seizure, TN/(TN + FP). The estimates of sensitivity and specificity can be applied directly to the annotation time series (time based assessment) or in an event based assessment (Fig. 2). The time based metrics correspond to an ‘overlap integral’ method of assessment (Wilson et al., 2003). The event based metrics correspond to an ‘any overlap’ method of assessment and must be modified so that specificity is replaced by a measurement of the false detections per hour (false positives per hour) due to a poorly defined ‘no seizure’ event (Wilson et al., 2003).The sensitivity and specificity can also be used to calculated the area under the receiver operator characteristic (a plot of the specificity vs the sensitivity). The effect of seizure duration on the accuracy of seizure detection (event based analysis) was also examined.

The assessment of agreement was examined on a case-by-case basis. Measures of agreement were then summarised across neonates using the median and interquartile range (the distribution of performance measures will be nonparametric). Agreement was assessed using Cohen’s Kappa index.

Performance metrics for the current validation study were also compared against results of the previous ‘leave one out’ study (Temko et al., 2013).

#### 2.2.2 Application specific measures of SDA usefulness

The agreement between several interpretations of the annotation was compared using the intra-class correlation coefficient (ICC). We quantify interpretation as a summary representation of clinically useful information on seizures over the entire EEG recording of a baby. This includes summary statistics such as seizure burden, seizure number, mean seizure duration, median seizure duration, seizure onset, and seizure period.

The ability of the SDA to support the identification of seizure and non-seizure babies was also examined, ie. detect any seizures in seizure babies and make no false detections in non-seizure babies.

Next the potential of the SDA to support clinical decisions regarding AED administration was examined. With periodic review of the EEG, seizures may not be detected immediately and AEDs are often administered some hours after seizure onset. AEDs may also be administered based on clinical assessment only, potentially erroneously. In order to facilitate this analysis, we examined whether there was seizure activity on the EEG in the 90 min prior to administration of AED (concurrent with AED), or absent in this 90 min period (non-concurrent with AED) to ascertain whether AED was given in a timely or appropriate manner. 90 min was taken as an arbitrary cut off time. This was compared to an examination of the SDA output to confirm whether AEDs had concurrent or non-concurrent SDA seizures. This comparison reflected the ability of an SDA to support clinical decisions regarding AED administration.

## 3 Results

In total, 107 babies recruited between 5th January 2009 and 30th June 2011 met the inclusion criteria (71 from CUMH and 26 from UCLH). A cohort of 70 babies was then formed by selecting all 35 who had EEG seizures and 35 babies who did not have EEG evidence of seizures. The 35 non-seizure babies were randomly selected from the recordings of the remaining 72 babies in order to match the number of seizure and non-seizure babies in the cohort. The range of demographics for this cohort of neonates is given in Table 3.

The seizure annotations by SM resulted in the detection of 2061 seizures in 35 neonates from a total of 4060 h of multi-channel EEG recordings (Table 4).

### 3.1 Conventional measures of agreement

Results of the comparison of seizure annotation by SM and GB produced a mean Kappa score of 0.851, which is considered near perfect. The level of agreement (time based analysis) between the annotations of the human expert (SM) and SDA at 9 SDA thresholds, are shown in Table 5A. The maximal level of agreement was at sensitivity threshold 0.4. Further time and event based measures assessed at each SDA threshold are shown in Table 5B.

**Fig. 3:**
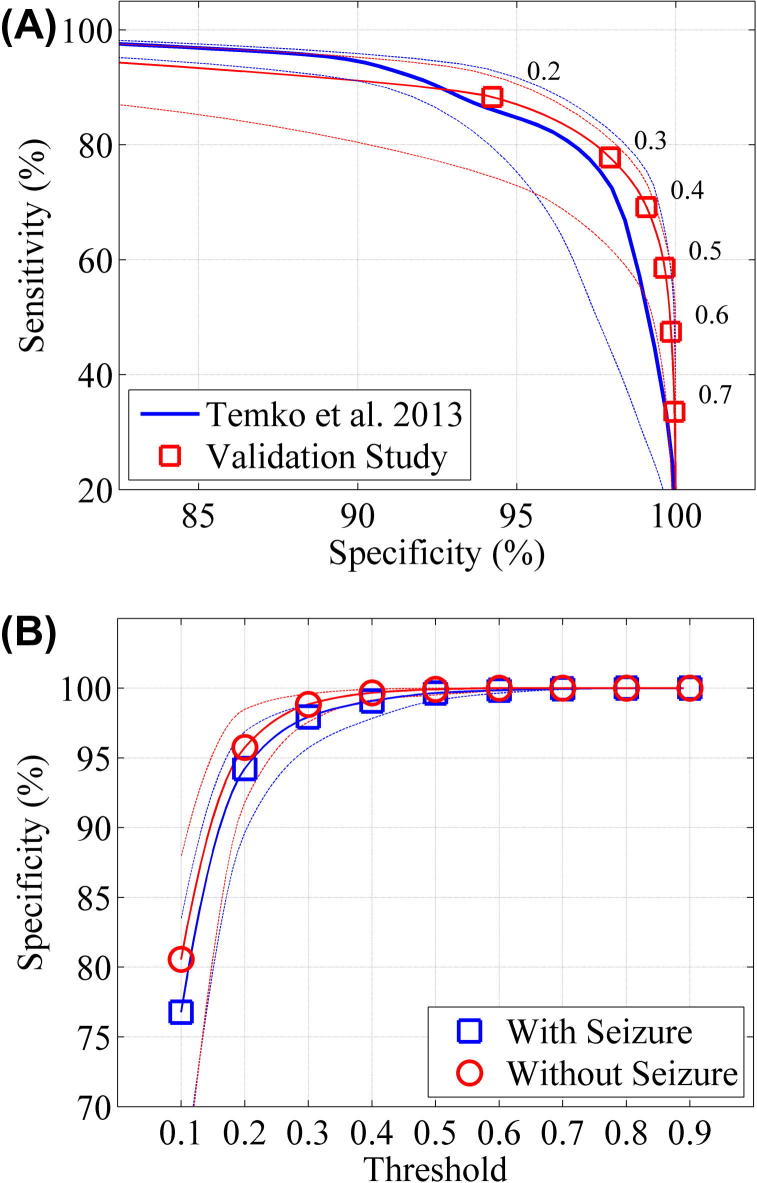
Time based measures (overlap integral) of SDA performance. The broken lines denote the interquartile range. (A) The median receiver operator curves (the trade-off between sensitivity and specificity) of validation set (estimated on babies with seizure, *N* = 35) compared to ‘leave one out’ cross validation set (Temko et al., 2013). The numbers on the plot relate to the threshold at which the sensitivity and specificity were estimated. (B) The median specificity of the SDA for validation set with respect to SDA threshold, estimated on babies with seizure (*N* = 35) and babies without seizure (*N* = 35) in validation study.

The results for time based metrics are also shown in Fig. 3. Fig. 3a compares the performance of the unseen validation study to the previous ‘leave one out’ cross validation (Temko et al., 2013). The median AUC for the validation study, estimated on neonates with seizures (sensitivity can only be estimated on neonates who have seizures) was 0.945 (IQR: 0.921–0.971, min: 0.684 max: 0.999). The mean AUC was 0.933. The performance curves for the two datasets are similar with slightly improved results in the validation set. Fig. 3b shows the specificity for neonates with seizure and neonates without seizure. The curves are similar with slightly higher specificity for non-seizure babies than seizure babies at lower thresholds.

**Fig. 4:**
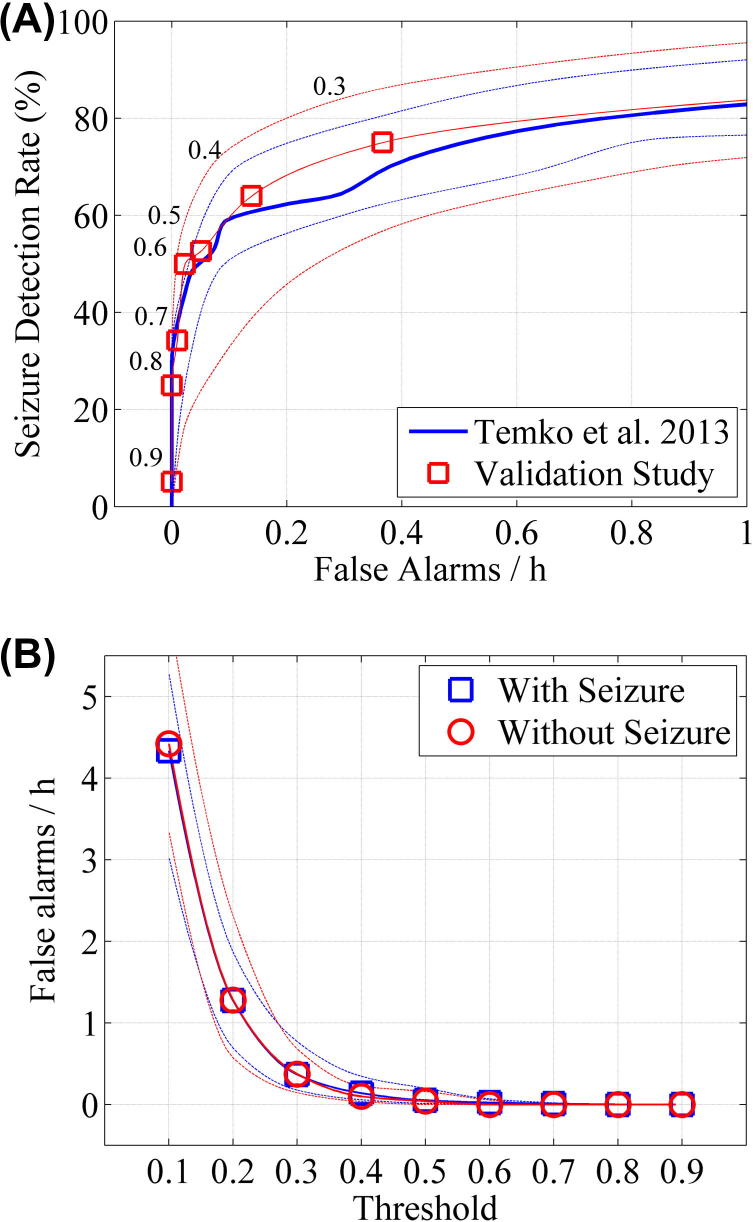
Event based measures (any overlap) of SDA performance compared to the original LOO cross-validation. The broken lines denote the interquartile range. (A) The trade-off between seizure detection rate and false alarms per hour for babies with seizure (*N* = 35) in validation study compared to ‘leave one out’ cross validation study (Temko et al., 2013). The numbers on the plot relate to the threshold at which the sensitivity and specificity were estimated. (B) The median false alarm rate with respect to SDA threshold estimated on babies with seizure (*N* = 35) and babies without seizure (*N* = 35) in validation study.

The results for event based metrics on a case by case basis are shown in Fig. 4. Again the performance curves for the validation study compared to the LOO cross validation are similar (Fig. 4a). Fig. 4b shows that false alarm rates are similar for seizure and non-seizure babies.

**Fig. 5:**
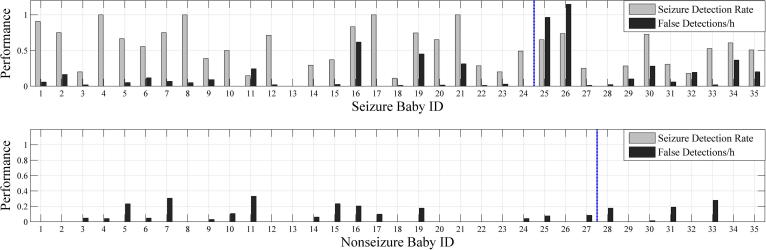
Seizure detection rates and FDs/h (SDA sensitivity threshold 0.5) for individual babies in the cohort. Note the high false detection rates in seizure babies 25 and 26 due to respiration and pulse artefact. Babies to the left of the vertical line were babies recruited in Cork and those to the right were recruited in London.

Fig. 5 shows seizure detection rates and FDs/h for individual babies in the cohort. There is variability in seizure detection and false detection rates across babies. Note the high false detection rates in seizure babies 25 and 26 due to respiration and pulse artefact.

**Fig. 6:**
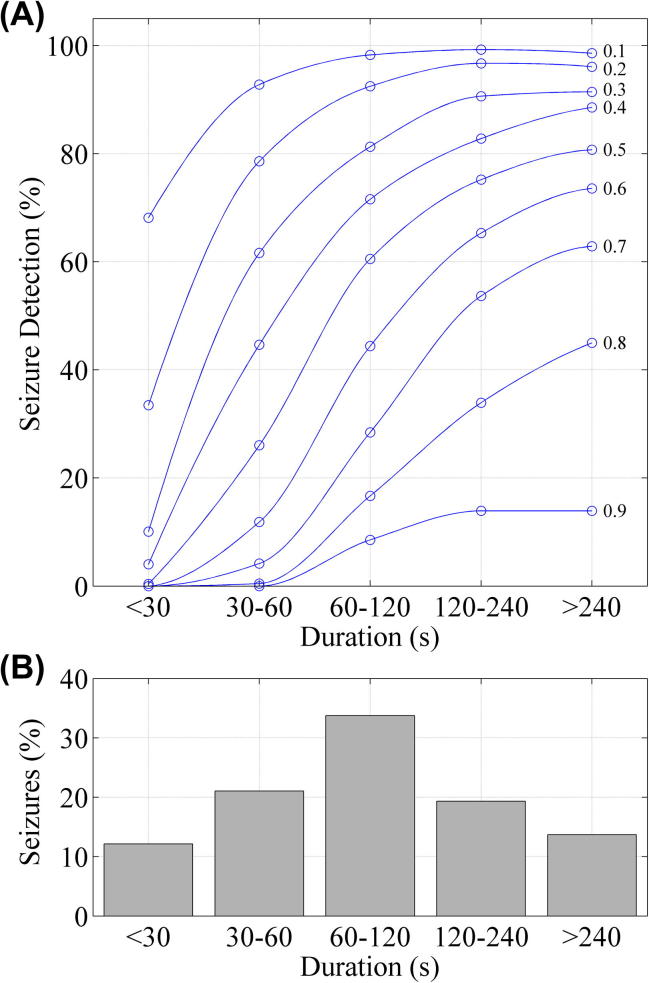
Analysis of seizure detection rate with respect to seizure duration. (A) SDA performance with respect to seizure duration over nine thresholds. (B) The distribution of seizure durations throughout the concatenated recording.

Fig. 6 shows the effect of seizure duration on SDA detection rate. The SDA performance is reduced when detecting short seizures. The most common seizure duration was 1–2 min.

#### 3.1.1 Application specific measures of SDA usefulness

The intra-class correlation between estimates of seizure burden, seizure number, mean/median seizure duration, seizure onset and seizure period are shown in Table 6 for the highest performing threshold of the SDA. The highest performing threshold varies depending on the parameter of interest.

**Fig. 7:**
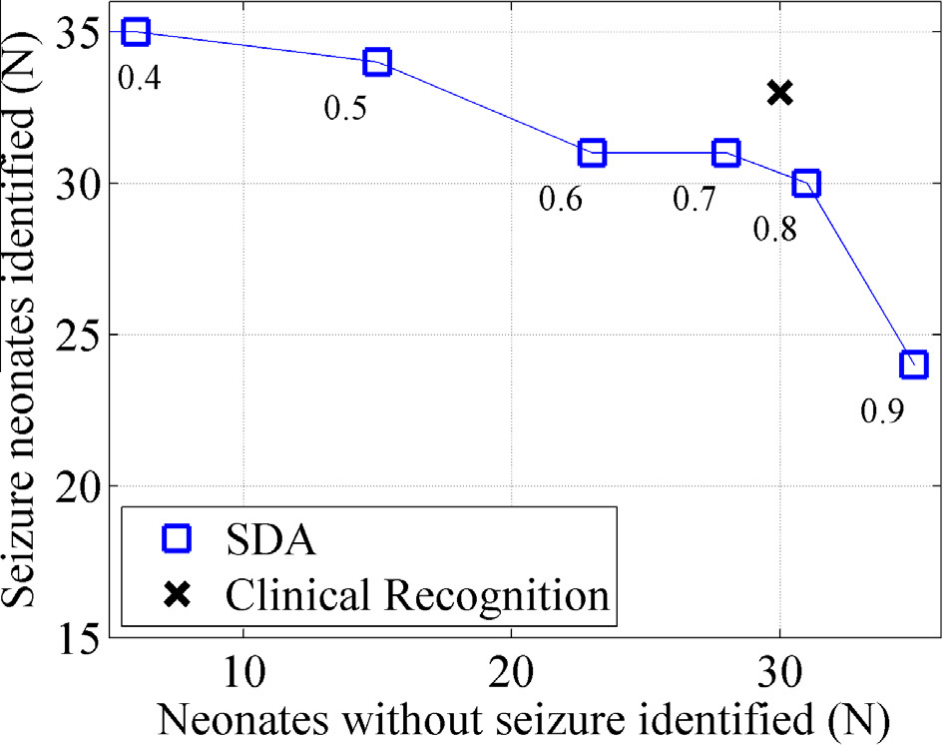
The accuracy of the SDA for the identification of seizure and non-seizure babies at several thresholds. There were 35 neonates with EEG evidence of seizure and 35 neonates without EEG evidence of seizure. Clinical recognition is based on AED administration and is superior to the SDA.

The performance of the SDA to support the identification of seizure babies (any seizure detected) and non-seizure babies (no false detections) at several clinically relevant thresholds is shown in Fig. 7. There is a trade-off between number of seizure and non-seizure babies detected depending on the SDA sensitivity threshold. The best performing SDA sensitivity threshold was at 0.8 (30/35 seizure babies identified, 31/35 non-seizure babies identified). Clinical recognition of seizure/non-seizure babies (identification of a seizure baby was assumed if AED was given, identification of a non-seizure baby was assumed if AED not given) was slightly superior to the SDA (33/35 seizure babies identified, 30/35 non-seizure babies identified). The SDA did not detect any seizures that had been missed by the expert reviewer in the non-seizure baby group.

**Fig. 8:**
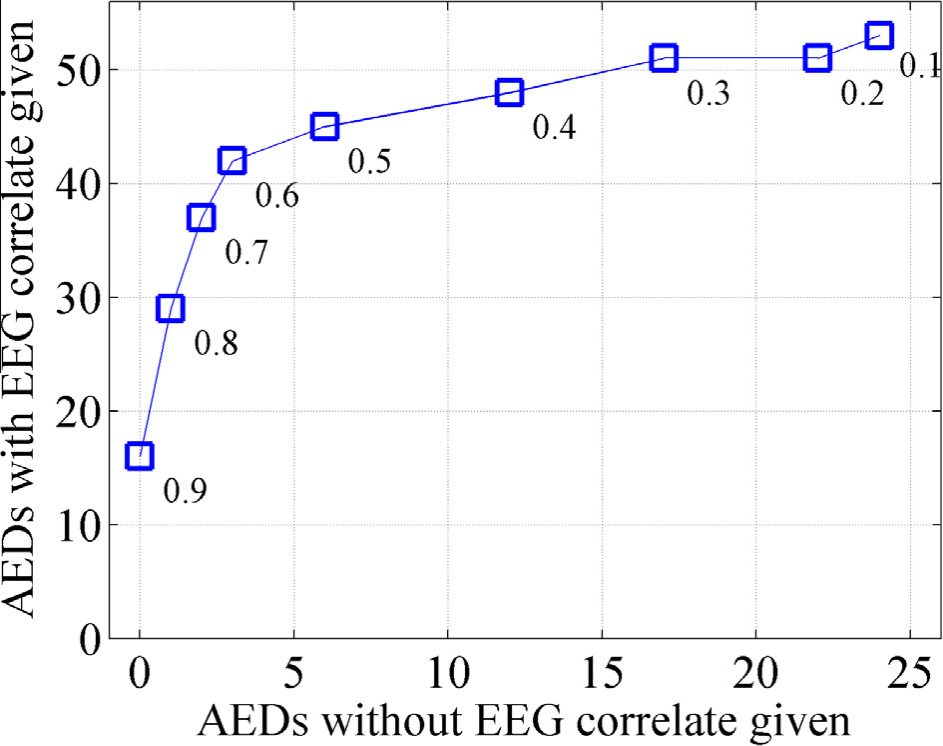
Potential of the SDA to support decisions on AED administration. A total of 97 AED administrations were recorded. Of these, 78 were administered concurrently with EEG recording and 53 were concurrent with EEG seizures (seizures occurring in the 90 min prior to AED adminstration). At a threshold of 0.5, 45 (85%) AED administrations concurrent with EEG evidence of seizure and only 6 (24%) of AED administrations with no EEG evidence of seizure would be supported by the SDA.

The potential of the SDA to support clinical decisions regarding AED administration is shown in Fig. 8. A total of 97 AED administrations were recorded (NB. Maintenance doses were not analysed). Of these, 78 were administered during EEG recording. 53/78 were concurrent with EEG seizures (within 90 min preceding AED administration) and 25/78 were administered with no concurrent seizures (in the 90 min preceding AED administration). Again there is a trade-off in the performance of the SDA to support clinical decisions regarding AED administration between supporting concurrent and non-concurrent AED decisions, dependent on SDA sensitivity threshold. The data does suggest however that there is a potential for the SDA to beneficially support these decisions. The SDA, at a threshold of 0.5 and 0.6 performed equally well in terms of its overall effectiveness to correctly identify seizures or seizure free EEG in the 90 min preceding AEDs and therefore to potentially support clinical decisions regarding AED administrations. At a threshold of 0.5, 45/53 (85%) AED administrations concurrent with EEG evidence of seizure would be supported by the SDA and only 6/25 (24%) of AED administrations with non-concurrent seizures would be supported by the SDA., ie. the SDA has the potential to reduce non-concurrent AED administration by 76% at a cost of not detecting 15% of concurrent seizures.

### 3.2 Missed seizures and false detections

**Fig. 9:**
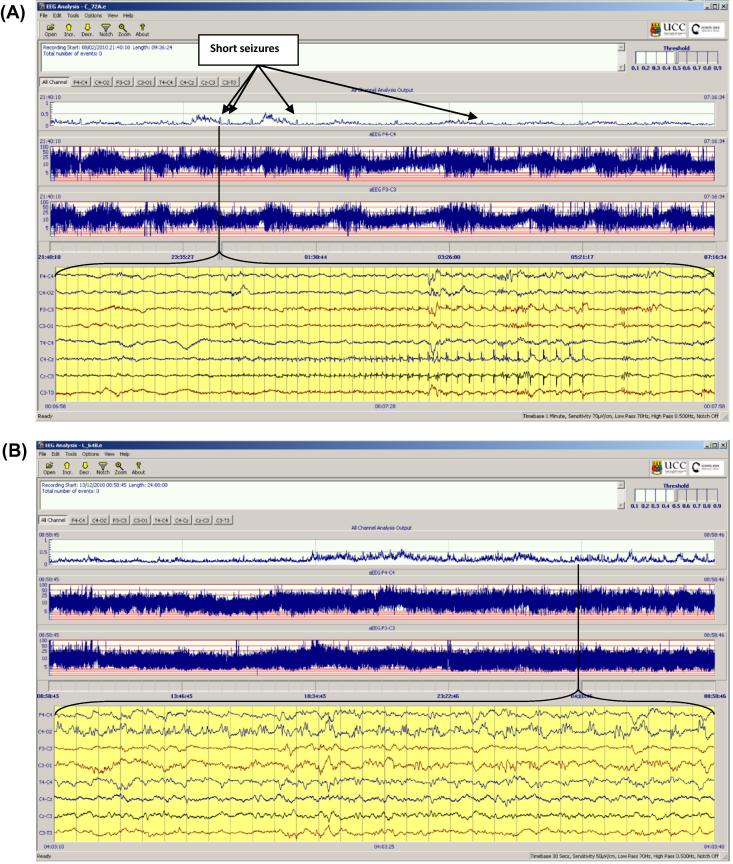

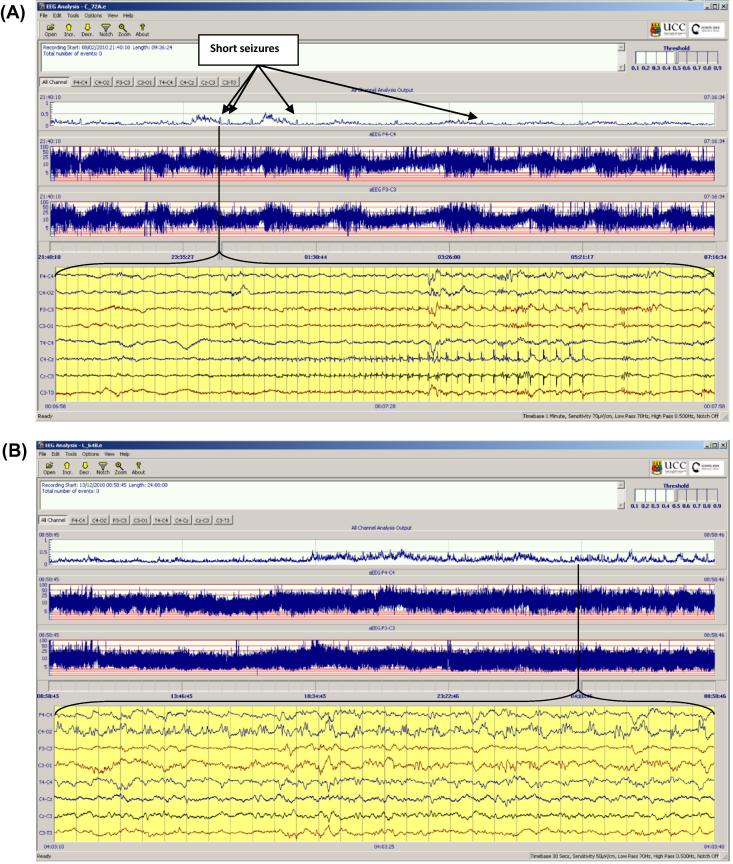

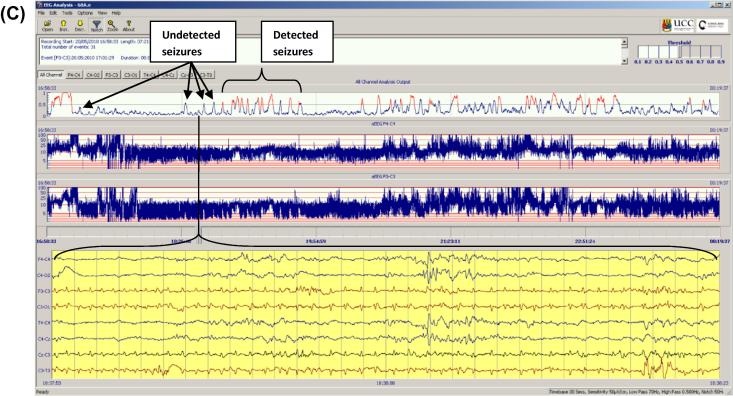
Seizures missed by the SDA. (A) Brief 30 s seizure. 0 of 4 seizures were detected in this record (thr 0.5), though the algorithm output would cause the clinician to interrogate the EEG at various points despite the fact that the fixed threshold was not reached. (B) Subtle, dysrhythmic 2 min seizure with complex morphology, 0 of 1 seizures were detected in this record (thr 0.5). (C) Low amplitude seizure, 31 of 55 seizures were detected in this record (note. Non detected seizures produce clear peaks on the probability trace for interrogation).

**Fig. 10:**
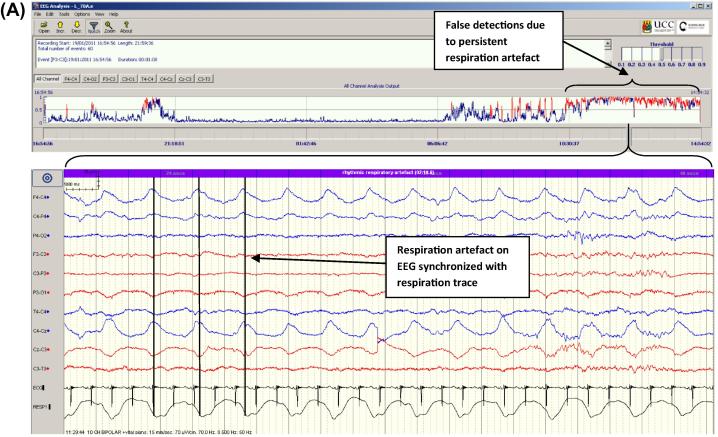

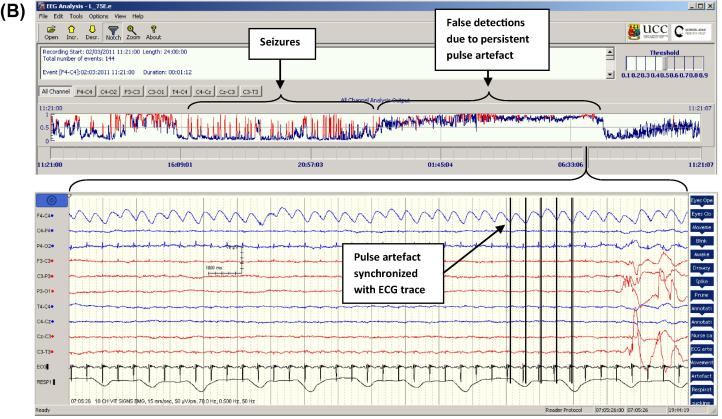

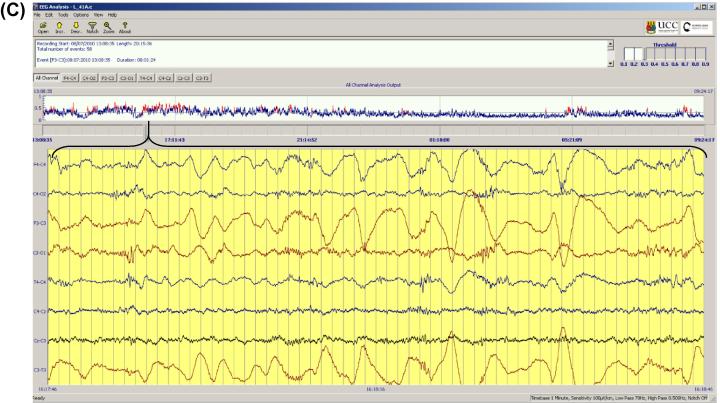

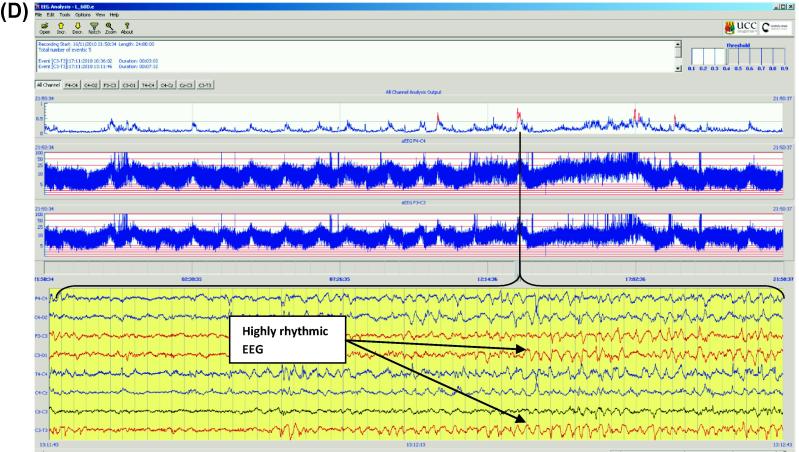
Causes of false detection. (A) Respiration artefact. Upper panel shows output from SDA, lower panel shows rhythmic respiration artefact on EEG synchronized with respiration trace (from motion sensor). (B) Pulse artefact synchronized to ECG trace. (C) Sweat artefact with characteristic high amplitude semi-rhythmic slow waves spanning several seconds. (D) Highly rhythmic background EEG occurring in the intermediate sleep phase. Note how periodic episodes of intermediate/quiet sleep indicated by the CFM are coincident with periods of raised seizure probability output on the SDA graph and a highly rhythmic EEG in the lower panel.

Examples of seizures that were not detected by the SDA are illustrated in Fig. 9. These were often short or low amplitude or had a dysrhythmic or complex morphology. A quantitative analysis of both missed seizures and false detections will be published separately. Some common causes of false detection are shown in Fig. 10. Respiration and pulse artefacts are recognisable as they are synchronized to the respiration and ECG traces respectively. Sweat artefact produces characteristic large semi-rhythmic waves spanning several seconds. A highly rhythmic background EEG pattern also caused false detections in some cases. This pattern was often observed in intermediate sleep, a phase between active and passive sleep, when widespread delta activity is known to increase. In some cases this delta activity had an increased rhythmicity than is commonly observed and consequently caused false detections. This is evident in Fig. 10d where the periodic peaks in the CFM indicating intermediate/quiet sleep correspond to peaks in the SDA probability output and a highly rhythmic EEG pattern is shown in the lower panel.

## 4 Discussion

In this study a comprehensive set of metrics have been used to measure the performance of our SDA on a large, unedited dataset of prolonged, clinical EEGs from two institutions. To the best of our knowledge this is the largest data set used for SDA validation in babies to date. Only a small subset of previous SDAs has been investigated on a large cohort of babies (Gotman et al., 1997, Lawrence et al., 2009, Mitra et al., 2009, Cherian et al., 2011).

We have used a reduced set of 9 recording electrodes in our study which the algorithm is preset to analyse. While some centres may favour a full set of electrodes (up to 32 recording electrodes) which are useful for the purposes of seizure onset localisation, our primary goal is seizure detection. A study by Tekgul et al. (2005) comparing seizure detection between a full 10:20 montage and a reduced 9 electrode set, found very few seizures were missed with a sensitivity of 96.8% for the reduced montage compared to the full set. The benefits of using more electrodes must be weighed against the time and technical constraints to the NNU staff of applying more electrodes out of hours.

We have found the performance of our algorithm to compare favourably with those previously reported by others (Table 1), although in previous papers not all metrics were reported for full comparison. For example Gotman (Gotman et al., 1997) reported a SDR of 66% with a FD rate of 2.3 FD/h. At a threshold of 0.3, the SDA reported here achieved a higher SDR of 75% at a much lower FD rate of 0.4 FD/h.

Navakatikyan (Navakatikyan et al., 2006), reported a SDR of 90% at 2 FD/h. In comparison, at a threshold of 0.2 our system achieved a slightly lower SDR rate of 85%, but with a much lower false detection rate of 1.2FD/h. In a clinical validation study of the Navakatikyan algorithm, Lawrence (Lawrence et al., 2009) compared the output of the algorithm with 12 h recordings of conventional video-EEG, and found a seizure detection rate of 55% with a false detection rate of 0.1 FD/h. This was quite different to the initial performance results (Navakatikyan et al., 2006). At a threshold of 0.5, the system reported here achieved a slightly lower SDR of 53% but again with a lower FD rate of 0.04/h.

Deburchgraeve et al. (2008) initially reported an SDR of 85% with a FD rate of 0.7FD/h. At a threshold of 0.2, our system achieved a similar SDR of 86% with a higher FD rate of 1.2 FD/h. However in a clinical validation of this algorithm, Cherian et al. (2011) reported a lower SDR rate of 66% with a FD rate of 0.58 FD/h. At threshold 0.4 our system achieved only a slightly lower SDR rate of 64.0% but with a considerably lower FD rate of 0.12 FD/h.

The SDA presented by Mitra et al. (2009) gave a SDR of 80% with a FD rate of 0.78 FD/h. The SDR is midway between the SDR rates reported here at threshold 0.3 (75.0%) and 0.2 (85%) with FD rates at 0.36 FD/h and 1.20 FD/h respectively, thus at 80% detection rate our FD rate would be comparable to that of Mitra.

In this study, the performance of our SDA on an unseen dataset mimicked the performance seen in the previous ‘leave one out’ cross validation (Temko et al., 2013). In ‘leave one out’ cross validation the data for each patient is tested using the data for all other patients as training data for the algorithm and the process repeated for each patient and the results averaged. These results suggest firstly that the data set used to train the algorithm contained a representative population of seizure and background EEG patterns and secondly that the SDA performs equally well on unseen data, as will be encountered in clinical use.

This study, in conjunction with the paper by Temko et al. (2013), are also the first papers to evaluate the SDA in a so-called ‘mismatch’ situation, where the seizure annotations of one expert are used to train the algorithm and the annotations of another expert are used to test the SDA. In addition, in this paper, we have, for the first time, tested the algorithm on data collected from two different centres, CUMH and UCLH, with potential differences in EEG application and recording. Given these two factors, the similarity of current SDA performance with previous performance demonstrates a practically acceptable degree of robustness of the algorithm.

The current performance, analysed with a very rigorous definition of true positive and true negative detections, was very good for most babies with seizures (Fig. 5). Two seizure babies (25 and 26) had high false detection rates due to respiration and pulsatile artefact. In future, it may be possible to teach clinical staff simple artefact “pattern” recognition (Fig. 10) so that false detection would not lead to overtreatment. For example, respiration and pulsatile artefact are both easily recognised as they are synchronized to the respiration and ECG traces respectively and are invariant as they do not show the evolving features of many seizures. Similarly sweat artefact produces characteristic high amplitude, semi-rhythmic slow waves spanning several seconds, a far slower frequency than typical seizures. Indeed, the results of the pilot study by Lawrence et al. in which pre-training was given, support this with only 1 single dose of AED given inappropriately in 232 false detection events (Lawrence et al., 2009).

The analysis of AED administration has shown that on 25 occasions AEDs were given without EEG seizures in the preceding 90 min and that the SDA has the potential to support clinical decisions to reduce AED administrations with ‘non-concurrent’ seizures. We are not suggesting that in 25 cases AEDs were given ‘inaccurately’ by clinical staff, In only one case did we identify that an AED had been given on clinical suspicion of seizure alone (without any EEG correlate at all). In most cases we suspect that there was simply a delay in detection of seizure and AED administration due to the nature of periodic EEG review which could potentially have been reduced with the support of the algorithm alerting clinical staff earlier.

The performance of the SDA has been presented over a range of sensitivity thresholds and the metrics used allow ‘best performing’ thresholds to be determined. However the choice of sensitivity threshold used in a clinical environment is critically dependent on the fact that best performing thresholds differ with tasks and threshold choice is therefore dependent on the requirement of the user. For example, the best performing threshold for detecting the maximal number of seizure/non-seizure babies correctly (Fig. 7) was threshold 0.8 while for supporting decisions regarding AED administration (Fig. 8), thresholds of 0.5/0.6 were optimal. The intra-class correlations in Table 6 show a variety of best performing thresholds for different parameters of interest. Notably, for detecting seizure onset and seizure number, a threshold of 0.4 performed best. For the task of correctly detecting the greatest ‘amount’ of seizure/non-seizure activity (seizure burden), time based analysis provides the most accurate measure and the Kappa score comparing human and SDA annotations indicated that the best performing threshold was also 0.4 (Kappa 0.630).

In clinical practice however, it is not likely that clinicians will be concerned with accurately detecting every single second of each seizure and are likely to care most that the SDA makes ‘some’ detection during a seizure and that overall the output of the SDA most accurately represents the numbers of seizures occurring with an acceptable false detection rate. This would allow treatment to be titrated to the presence of ongoing seizures, and in this respect the event based metrics may be of more interest clinically. The concept of what is deemed *acceptable* in terms of the rate of false alarms is also dependent on user preference and may vary between users, affecting the choice of sensitivity threshold.

We consider the output from the SDA at thresholds from 0.5 to 0.3 to be within a clinically acceptable range, giving detection rates between 52.6% and 75.0% with false detections, on average, approximately every 20 and 3 h respectively (Table 5B). This range is proposed on the basis of a perceived expectation that a minimum of 50% seizure detection is required and that a false detection rate of greater than 0.5/h might be considered excessive.

The data presented here represents only one stage in the assessment of the SDAs performance which will be further tested in a ‘live’ multicentre randomised clinical evaluation (the ANSeR study – Algorithm for Neonatal Seizure Recognition http://clinicaltrials.gov/show/NCT02160171). For this study the threshold will be preset at 0.5 for purposes of equivalence across participating centres.

It is important to state that the SDA is not intended to replace clinician’s review of the EEG or to be viewed as a ‘decision maker’ with regard to the presence, or not, of seizures. Its purpose is only to highlight areas of interest for further review. In this respect, a crucial aspect of the algorithm’s output is the graph of the probability of seizure. A clinician reviewing the output of the SDA at the cotside is likely to interrogate both prominent peaks that breach the threshold on the graph and others that do not (eg. Fig. 9c). With a “pattern recognition” support package, the ability of clinicians to differentiate seizures from artefacts can, potentially, be improved. For these reasons, the role of the reviewer is central to the interpretation of the output of the SDA and consequently how many seizures, false detections, seizure babies and non-seizure babies are identified correctly. Our intention is that the seizure detection performance of clinicians with the assistance of our algorithm will be superior to the algorithm’s simple binary ‘alerts’ based on fixed thresholds presented here.

## 5 Conclusion

We have validated a neonatal SDA on a large EEG dataset and have shown that it achieves a clinically useful level of seizure detection with acceptable false detection rates. Future multi-centre evaluation of the SDA in a ‘live’ clinical environment will critically investigate the clinician’s interpretation of the full SDA output to determine the usefulness of the SDA in the NICU.

## Figures and Tables

**Table 1:** Summary of SDAs proposed in the literature. (DB – database, h – hour, S – seizure, NS – non-seizure, Dur – duration, AUC – area under the receiver operator characteristic, Sens – sensitivity, spec, specificity, SDR – seizure detection rate, FA/h – false alarms per hour).

Algorithm	DB length h (*N*)	S:NS Dur	NS neonates	AUC	Sens (%)	Spec (%)	SDR (%)	FA/h (N/h)
Liu et al. (1992)	1.0 (14)	1:1	Yes		84	98		
Gotman et al. (1997)	237 (54)		Yes				66	2.3
Smit et al. (2004)	10.4 (19)		No		66	90		
Navakatikyan et al. (2006)	24 (55)	1:6.8	Yes		83	87	90	2
Lawrence et al. (2009)	2708 (40)		Yes				55	0.09
Deburchgraeve et al. (2008)	218 (26)		Yes				85	0.66
Cherian et al. (2011)	756 (24)	1:27.9	No		59		66	0.58
Mitra et al. (2009)	120 (76)	1:11.0	Yes				80	0.78
Temko et al., 2011a, Temko et al., 2011b	268 (17)	1:5.9	No	0.96	90	90	89	1
Temko et al. (2013)	2540 (51)		Yes	0.96			71	0.25

**Table 2:** Main features extracted from the EEG by the SDA.

Groups	Feature list
Frequency domain	• Total power (0–12 Hz) • Peak frequency of spectrum • Spectral edge frequency (80%, 90%, 95%) • Power in 2 Hz width sub-bands (0–2 Hz, 1–3 Hz, ...10–12 Hz) • Normalised power in sub-bands • Wavelet energy (the EEG is decomposed into 8 coefficients using the Daubechy 4 wavelet, the energy in the 5th coefficient corresponding to 1–2 Hz is used as a feature)

Time domain	• Curve length • Number of maxima and minima • Root mean squared amplitude • Hjorth parameters • Zero crossings (raw epoch, Δ, ΔΔ) • Autoregressive modelling error (model order 1–9) • Skewness • Kurtosis • Nonlinear energy • Variance (Δ, ΔΔ)

Information theory:	• Shannon entropy • Singular value decomposition entropy • Fisher information • Spectral entropy

**Table 3:** Demographics and EEG recording information relating to the 70 neonates used in this study.

Gestational age (weeks^+days^)†		40^+3^ (39^+2^ to 41^+2^)	
Birthweight (g)†		3526 (3140 to 3920)	
Gender (male:female)		37:33	
Age at EEG onset (h)†		7.0 (3.6–19.0)	
EEG recording duration (h)†		51.6 (21.5–84.4)	

Primary diagnoses		Neonates (N)	

HIE^a^		37	
	Mild		10
	Moderate		19
	Severe		8
Birth depression^b^		10	
Stroke^c^		8	
Focal lesion^d^		3	
Other^e^		12	

† Median (interquartile range).

a 22 treated with therapeutic hypothermia (TH).

b Birth depression without ensuing encephalopathy.

c Stroke – arterial ischaemic, haemorrhagic, multiple infarctions.

d Focal lesion – subdural haemorrhage, intraparenchymal bleed.

e Other – meningitis/HIE (TH), viral encephalitis, sepsis, benign familial neonatal seizures, benign sleep myoclonus, unknown diagnosis.

**Table 4:** The summary of seizure characteristics in the 35 babies with EEG confirmation of seizures. Seizure onset (h) refers to post natal age in hours.

	Median	IQR	min	max
Seizure onset (h)	19.0	(11.5–35.8)	6.6	153.8
Seizure period (h)	18.6	(8.6–35.4)	0.03	120.2
Seizure burden (mins)	79.8	(25.3–204.6)	1.9	1404
Seizure number (*N*)	22	(7–75)	1	295
Mean seizure duration (s)	163	(95–298)	28	2207
Median seizure duration (s)	115	(69–186)	25	2207

**Table 5:** The level of agreement between the annotation of the human expert (SM) and the SDA at 9 thresholds. (A) Cohen’s Kappa Index (time based metric), (B) sensitivity and specificity (time based metric), seizure detection rate and false alarms per hour (event based metrics). Data are median (IQR).

SDA threshold	Kappa^a^	Prevalence index^a^	Bias index^a^
*(A)*			
0.1	0.098 (0.044–0.204)	0.688 (0.588–0.780)	0.222 (0.157–0.364)
0.2	0.309 (0.155–0.509)	0.893 (0.785–0.949)	0.050 (0.027–0.098)
0.3	0.524 (0.217–0.677)	0.936 (0.862–0.977)	0.011 (0.003–0.031)
0.4	0.630 (0.283–0.739)	0.956 (0.885–0.984)	0.006 (0.003–0.019)
0.5	0.579 (0.332–0.724)	0.954 (0.896–0.983)	0.007 (0.002–0.019)
0.6	0.552 (0.340–0.700)	0.952 (0.864–0.981)	0.006 (0.003–0.019)
0.7	0.405 (0.255–0.621)	0.959 (0.879–0.983)	0.001 (0.003–0.027)
0.8	0.280 (0.135–0.406)	0.957 (0.889–0.989)	0.011 (0.004–0.038)
0.9	0.060 (0–0.207)	0.941 (0–0.981)	0.007 (0–0.042)

SDA threshold	Sensitivity^a^	Specificity^b^	Seizure detection rate^a^	False alarm rate^b^
*(B)*				
0.1	96.2 (92.6–98.4)	77.7 (63.0–86.6)	97.1 (92.2–100.0)	4.35 (3.28–5.70)
0.2	88.1 (80.5–92.3)	94.5 (91.1–97.9)	85.7 (77.1–97.6)	1.20 (0.63–2.01)
0.3	76.1 (68.5–83.9)	98.5 (97.0–99.5)	75.0 (59.5–91.7)	0.36 (0.16–0.74)
0.4	68.6 (56.3–80.2)	99.5 (98.6–99.9)	64.0 (41.6–85.3)	0.12 (0.04–0.29)
0.5	58.6 (41.2–70.4)	99.8 (99.3–100.0)	52.6 (28.3–73.4)	0.04 (0–0.18)
0.6	44.2 (31.4–70.0)	99.9 (99.7–100.0)	50.0 (23.1–60.1)	0 (0–0.06)
0.7	32.1 (15.0–51.0)	100.0 (99.9.0–100.0)	34.2 (13.4–50.0)	0 (0–0.01)
0.8	16.4 (7.4–30.9)	100.0 (100.0–100.0)	23.7 (7.2–38.9)	0 (0–0)
0.9	3.4 (0–12.1)	100.0 (100.0–100.0)	5.1 (0–16.2)	0 (0–0)

a Estimated on neonates with seizure (*N* = 35).

b Estimated on all neonates (*N* = 70).

**Table 6:** Agreement between interpretation of annotations of the human expert (SM) and the SDA. Interpretation relates to summary statistics of the temporal evolution of seizures in each EEG recording. The average intra-class correlation coefficient (ICC) is presented and is estimated on the entire cohort of babies (*N* = 70). The summary statistics of seizures are estimated from the SDA annotation but only on babies who have seizure as detected by the human expert. The threshold that results in the highest ICC for each measure is shown. The SDA detects at least one seizure in 64, 54, 43, 38 babies at thresholds of 0.4, 0.5, 0.6, 0.7 respectively.

	Median	IQR	ICC	SDA threshold
Seizure onset (h)	15.1	(7.7–32.5)	0.722	0.4
Seizure period (h)	26.3	(7.3–49.8)	0.802	0.6
Seizure burden (mins)	81.1	(26.6–181.3)	0.859	0.5
Seizure number (*N*)	35	(7–72)	0.930	0.4
Mean seizure duration (s)	169	(121–243)	0.511	0.5
Median seizure duration (s)	124	(92–146)	0.323	0.7
